# Beyond the victim: experienced and observed workplace violence, violence prevention, and job dissatisfaction

**DOI:** 10.3389/fpsyg.2026.1870141

**Published:** 2026-07-13

**Authors:** Emily Belew, Madelynn Stackhouse

**Affiliations:** 1 Department of Management, Bryan School of Business and Economics, UNC Greensboro, Greensboro, NC, United States; 2 Department of Public Health, Texas College of Osteopathic Medicine, UNT Health, Fort Worth, TX, United States

**Keywords:** job attitudes, job satisfaction, psychological contract violation, transgressions, workplace violence

## Abstract

**Introduction:**

Workplace violence rarely only affects its direct victims; it is often witnessed, remembered, and silently interpreted by many others. This study explores the impacts of experienced and observed violence on job dissatisfaction and the mediating effects of perceived lack of organizational violence prevention (LOVP).

**Methods:**

Data were drawn from a nationally representative sample of 14,515 federal employees. Structural equation modeling was used to test direct, indirect, and comparative effects.

**Results and discussion:**

The results indicated that first-person and observed third-person violence result in job dissatisfaction. Interestingly, the contrast between first-person (experienced) and third-person (observed) violence on job dissatisfaction is negligible. Both exposure types have significant total effects (0.47 versus 0.33), respectively, but the difference is non-significant (*p* = 0.182). Similarly, while LOVP fully mediated the link between both experienced or observed violence and job dissatisfaction, the difference in indirect effects through LOVP is also non-significant (*p* = 0.151). This study demonstrates that job dissatisfaction does not differ significantly between firsthand and observed exposure, challenging assumptions about the primacy of direct victimization. Further, by showing that perceived lack of organizational violence prevention (LOVP) fully mediates these relationships, we reveal why both forms of exposure equally erode employee attitudes: employees interpret violence as an organizational failure rather than an isolated act. This perspective reframes workplace violence as a relational and organizational phenomenon rather than a purely stress-based event.

## Introduction

Workplace physical violence threatens employees globally, prompting urgent policy responses such as the International Labour Organization’s Convention 190 (International Labour Organization, 2024), the fastest ratified convention in the past decade. Magnified by the COVID-19 pandemic, workplace *physical* violence, though less prevalent than psychological forms of harm like ostracism, bullying, or other types of aggression (e.g., Antón et al., 2022; Hershcovis, 2011; Howard et al., 2020), poses a significant threat to the stability and well-being of workers across multiple critical sectors, such as healthcare, law enforcement, and retail (Bushell and Braithwaite, 2024; Lu et al., 2023; Rossi et al., 2023; Zhang et al., 2024). Moreover, prior studies demonstrate that direct physical victimization is more damaging than witnessing physical violence (Michael et al., 2016; Zhou et al., 2017), though recent mistreatment observer research highlights the surprisingly intense psychological effects of observation experiences (Hill et al., 2025). Prompted by both this urgency and persistent ambiguity surrounding exposures and pathways to harm, greater clarity regarding distinct forms of workplace violence and their consequences for job-related attitudes is warranted.

Measures of physical workplace violence often conflate various forms of harm to include threats of violence, psychological aggression, and vicarious violence (Heponiemi et al., 2014; Mueller and Tschan, 2011; Rogers and Kelloway, 1997; Schat and Kelloway, 2000). Our study isolates physical violence from these other forms and also distinguishes between *experienced (firsthand)* and *observed (third-party)* exposure to physical violence. Drawing on psychological contract and social exchange theories (Cropanzano et al., 2017; Robinson and Rousseau, 1994), we examine how perceptions of organizational prevention—or lack thereof—shape employees’ responses. Rather than assuming that attitudes stem solely from experiencing the violent act, our approach considers what violence communicates about the employer’s obligations. Does witnessing violence signal the same breach of trust as experiencing it? Does organizational prevention buffer these effects, or does its absence amplify them? By exploring these questions in a large sample of 14,515 employees, we re-center workplace violence as a relational and organizational phenomenon, moving beyond stress-based explanations toward a deeper understanding of how meaning-making processes drive job attitudes.

Our study advances theory in several ways. First, by empirically disentangling physical violence from other forms of workplace aggression and by distinguishing experienced from observed exposure (LeBlanc and Kelloway, 2002), we reopen a question that prior work has largely left implicit (Heponiemi et al., 2014; Mueller and Tschan, 2011; Schat and Kelloway, 2000): whether proximity to harm or the interpretation of harm shapes job attitudes. This separation sharpens theoretical accounts of how form and encounter with violence map onto dissatisfaction, without presuming that immediacy alone determines impact.

Second, we shift the lens from stressor–strain explanations (Barling, 1996; Michael et al., 2016; Mueller and Tschan, 2011; Zhou et al., 2017) toward relational interpretations grounded in Social Exchange Theory and Psychological Contract Theory (Cropanzano et al., 2017; Robinson and Rousseau, 1994). In this view, violence is not merely an event; it is a signal of whether the organization honors its implicit promise of safety and reciprocity. When that promise falters, employees recalibrate their attitudes consistent with processes of contract breach and exchange imbalance (Cropanzano et al., 2017; Robinson and Rousseau, 1994).

Third, we identify perceived lack of organizational violence prevention (LOVP) as the mechanism through which exposure to physical violence shapes job dissatisfaction, thereby reframing harm as a response to organizational failure rather than solely to the act itself. This perspective highlights how perceived prevention (or its absence) communicates neglect, activates breach-related cognitions, and precipitates withdrawal and dissatisfaction for employees whether they were the target or witness, thus challenging assumptions about the primacy of direct victimization. Considered together, the present study demonstrates that job dissatisfaction following extreme workplace violence is driven not by proximity to harm, but by shared evaluations of organizational prevention failure.

## Theoretical framework and hypotheses

One of the most fundamental expectations employees hold is that their organization will ensure physical safety at work (Newaz et al., 2019; Walker and Hutton, 2006; Walker, 2013). This expectation is embedded in the psychological contract, defined as the implicit understanding that employers will provide a secure environment in exchange for employee contributions (Robinson, 1996; Rousseau, 1995). When physical violence occurs, whether directly experienced or witnessed, that expectation is threatened—called a “breach” in psychological contract theory (PCT; Robinson, 1996).

Complimenting this view is a social exchange theory perspective (SET; Blau, 1964/1986; Cropanzano et al., 2017). Workplace relationships are built on reciprocal exchanges, where employees and organizations provide resources and support to one another over time. Employees evaluate these exchanges based on the organization’s fulfillment of its responsibilities, particularly the ability to foster a supportive and just work environment (Cropanzano et al., 2017; Parzefall and Salin, 2010). Emphasizing reciprocity, when organizations uphold these obligations, employees respond with engagement, satisfaction, and discretionary effort. Alternatively, unmet obligations trigger withdrawal, dissatisfaction, or disengagement (Cropanzano et al., 2017; Robinson, 1996).

**Figure 1:**
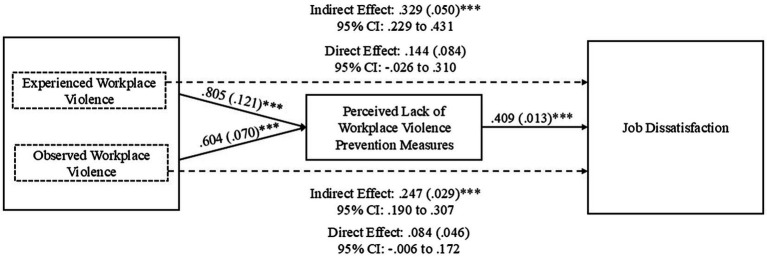
Conceptual model of experienced and observed workplace violence predicting job dissatisfaction via perceived lack of organizational violence prevention (LOVP). Dotted lines indicate non-significant paths. Unstandardized regression coefficients (*B*) are reported. ***p < 0.001. Confidence interval based on 5,000 bootstrap resamples.

Together, Social Exchange Theory (SET) and Psychological Contract Theory (PCT) provide a coherent framework for understanding how workplace violence undermines employee–organization relationships. SET emphasizes the relational consequences of disrupted exchanges, while PCT specifies employee expectations as perceived contractual obligations. Integrating these perspectives, we argue that violence functions less as a discrete stressor and more as a relational signal: it communicates whether the organization honors its implicit promise of protection. That is, our model emphasizes workplace physical violence as initiating a meaning-making mechanism (see Figure 1). When prevention is perceived as strong, employees infer adherence to the safety contract; when prevention appears inadequate, they infer neglect and breach (Walker and Hutton, 2006), thereby translating exposure into job dissatisfaction through perceived lack of organizational violence prevention (LOVP).

### Experienced vs. observed violence: competing perspectives

Workplace violence research suggests that both directly experienced and witnessed physical violence can undermine employee well-being and job-related attitudes, including job dissatisfaction and intent to leave (Duan et al., 2019; Dupré et al., 2014; LeBlanc and Kelloway, 2002). However, many prior studies have measured these forms together (Mueller and Tschan, 2011; Rogers and Kelloway, 1997) or modeled them as a single construct (Dupré et al., 2014), leaving the relative impact of firsthand versus observed violence unclear. While some research provides separate estimates for direct and vicarious exposure to physical violence, these effects are often examined through mediators such as fear of future violence and the relative impact is not considered (Mahmut et al., 2021; Dupré et al., 2014; Mueller and Tschan, 2011).

Stress-based models suggest that direct exposure should exert stronger psychological and work-related consequences than indirect exposure (Michael et al., 2016; Zhou et al., 2017), particularly for high-intensity acts like physical assault (May and Wisco, 2016). From this view, firsthand violence represents a personally salient breach, likely to provoke greater dissatisfaction (Johnson and O’Leary-Kelly, 2003).

Conversely, emerging research on mistreatment observers reveals that witnessing violence can be surprisingly harmful (Hill et al., 2025). Observers may experience identity threat, injustice perceptions, and fear of future harm (David et al., 2024; Leigh and Melwani, 2022). These reactions can erode trust and job attitudes, especially when organizational responses appear indifferent or ineffective (Dhanani and LaPalme, 2019). In some cases, vicarious experiences may even exceed direct experiences in shaping attitudes, as suggested by incivility research where group-level exposure predicted job satisfaction more strongly than direct incivility experience (Lim et al., 2008).

Finally, it is plausible that both exposure types exert similar effects on job dissatisfaction. If employees interpret violence primarily as an organizational failure to uphold safety obligations (Walker and Hutton, 2006; Walker, 2013), then the distinction between experiencing and observing may matter less than previously assumed. This possibility challenges conventional assumptions and underscores the need for empirical testing. We therefore propose alternative hypotheses:

*Hypothesis 1a (Directional – Experienced stronger):* Employees who directly experience physical violence will report greater job dissatisfaction than those who witness violence.

*Hypothesis 1b (Alternative – Observed stronger):* Employees who witness physical violence will report greater job dissatisfaction than those who directly experience it.

*Hypothesis 1c (Null – Non-directional):* There will be no significant difference in job dissatisfaction between employees who directly experience physical violence and those who witness it, suggesting that both exposure types similarly undermine job attitudes.

### The mediating role of organizational violence prevention efforts

Drawing on the integrated psychological contract social exchange framework above, we treat physical violence efforts as a pivotal interpretive bridge between violent incidents and employee attitudes. In this view, LOVP (perceived lack of organizational violence prevention) is not merely an environmental condition but an attributional mechanism: it shapes whether employees construe violence as an isolated anomaly or as evidence that the organization has failed to honor its implicit safety obligation. When prevention appears credible and visible *vis-a-vis* clear policies, prompt investigations, accessible reporting channels, and resources for affected employees, workers infer adherence to the safety contract. This preserves trust and reciprocity in the exchange relationship (Cropanzano et al., 2017; Walker and Hutton, 2006; Walker, 2013). Conversely, when prevention is perceived as inadequate *vis-a-vis* ambiguous procedures, inconsistent enforcement, or indifferent managerial responses, employees interpret the event as organizational neglect, triggering breach cognitions and withdrawal-oriented attitudes such as job dissatisfaction (Parzefall and Salin, 2010; Robinson, 1996; Rousseau, 1995). Conceptually, LOVP thus operates as the meaning-making conduit that translates exposure (experienced or observed) into attitudinal outcomes, aligning the safety obligation specified by PCT with the reciprocity logic emphasized by SET.

This mediational logic also clarifies why proximity to harm may not be the decisive factor for job dissatisfaction. Whether employees are targets or witnesses, the salient question within the relationship is what the organization did—or failed to do—before, during, and after the incident. If prevention signals are weak, both targets and observers may update their beliefs about the employer’s reliability and justice orientation, leading to comparable decrements in satisfaction (Bianchi et al., 2023; David et al., 2024; Leigh and Melwani, 2022). Positioning LOVP at the center of the model therefore extends stressor–strain accounts by reframing violence as a relational signal rather than solely a physiological or emotional stressor leading to direct (i.e., mood and fear) and indirect outcomes (i.e., turnover) (Barling, 1996; Dupré et al., 2014; LeBlanc and Kelloway, 2002; Mueller and Tschan, 2011). It also integrates safety-contract scholarship with exchange-based theorizing, specifying why exposure, regardless of type, erodes job attitudes. Not only is this because violence is harmful, but because employees construe insufficient prevention as a breach of the most basic duty of care (Walker and Hutton, 2006; Walker, 2013). Empirically, this perspective motivates tests of indirect effects through LOVP and contrasts across exposure types, adjudicating whether differences in dissatisfaction reflect proximity to harm or common breach interpretations anchored in organizational prevention.

Thus, LOVP serves as the psychological mechanism through which violence exposure translates into job dissatisfaction. On one hand, experienced violence signals breach of the safety contract: employees attribute dissatisfaction to organizational prevention failure rather than the incident per se. Specifically:

*Hypothesis 2: LOVP* mediates the positive relationship between firsthand (experienced) workplace physical violence and job dissatisfaction.

On the other hand, witnessing violence can trigger breach attribution appraisals when prevention appears inadequate, producing comparable attitudinal consequences.

*Hypothesis 3:* LOVP mediates the positive relationship between third-party (observed) workplace physical violence and job dissatisfaction.

Grounded in Social Exchange Theory and Psychological Contract Theory (Blau, 1964/1986; Cropanzano et al., 2017; Robinson, 1996; Rousseau, 1995), we expect that employees interpretations of organization prevention carry the effect of workplace physical violence to job dissatisfaction. If perceived lack of organizational violence prevention (LOVP) is the core breach signal, both targets and witnesses should arrive at similar attitudinal conclusions when prevention appears inadequate. Accordingly, our primary contrast hypothesis posits parity in the mediated effects across exposure types, with directional alternatives retained to adjudicate competing stressor–strain accounts. The null contrast on the mediated path posits that what drives job dissatisfaction is not proximity to harm but the interpretation of organizational prevention. Whether employees are targets or witnesses, violence communicates information about the employer’s reliability, justice orientation, and adherence to the implicit safety obligation; when prevention is perceived as inadequate (LOVP), both groups infer breach and exchange imbalance, producing comparable attitudinal consequences (Blau, 1964/1986; Cropanzano et al., 2017; Robinson, 1996; Rousseau, 1995).

Indeed, prior work shows that observation can be a surprisingly potent psychological event, eliciting identity threat and injustice appraisals similar to those triggered by direct victimization (David et al., 2024; Hill et al., 2025), and that group-level mistreatment can depress job satisfaction equal to or even more than personal exposure (Lim et al., 2008). Safety-contract research further underscores that employees anchor their judgments in organizational prevention signals like visible policies, consistent enforcement, and responsive support, so perceived prevention failure functions as a meaning-making mechanism across exposure types (Walker and Hutton, 2006; Walker, 2013). Thus, if LOVP fully carries the effect of violence to dissatisfaction, the indirect effects for experienced and observed exposure should not differ significantly, because both hinge on the same breach-based appraisal rather than on the immediacy of the harm.

*Hypothesis 4a (null non-directional):* The indirect effect of workplace physical violence on job dissatisfaction via perceived lack of organizational violence prevention (LOVP) will not differ significantly between employees who experience violence and those who observe it.

We also offer directional alternatives retained for alternative accounts:

*Hypothesis 4b (Directional—Experienced stronger):* The indirect effect via LOVP will be stronger for employees who experience violence than for those who observe it.

*Hypothesis 4c (Alternative—Observed stronger):* The indirect effect via LOVP will be stronger for employees who observe violence than for those who experience it.

## Materials and methods

### Participants

The data for this study were drawn from the 2016 United States Merit Principles Survey Path 1 dataset (MPS) (U.S. Merit Systems Protection Board, 2016a,b). This nationally administered survey assesses federal employees’ perceptions of workplace practices, management, and organizational climate. Federal workers face similar or greater accounts of physical violence than private-sector employees and include high risk roles like correction or healthcare workers (Harrell, 2013; U.S. Merit Systems Protection Board, 2012).

The MPS, a cross-sectional survey of a stratified random sample of federal full-time employees, was distributed electronically between July and September 2016 to approximately 114,000 employees (U.S. Merit Systems Protection Board, 2016a). Participation was voluntary and confidential, and the final dataset was deemed representative of the federal workforce by the Merit Systems Protection Board (U.S. Merit Systems Protection Board, 2016a). The MPS had an estimated margin of error of 1.1% (95% CI = 0.30–8.80%). Measurement of key constructs is broken between sections and involves different measurement scales, reflective of practices designed to reduce common method variance (CMV) (Podsakoff et al., 2024).

Of the 14,515 employees who completed the Path 1 survey, cases with missing data on key variables were excluded. The final analytic sample for the structural equation model (SEM) consisted of 10,608 complete cases. Because the regression models included a smaller set of variables, they retained a larger number of observations due to fewer missing data requirements (see Table 2 for regression model-specific sample sizes). Within the SEM sample, 96 employees (0.90%) reported first-hand experience of physical workplace violence, and 233 employees (2.20% reported observing physical violence toward others). The remaining participants (*n* = 10,279) reported no exposure to physical workplace violence. This group served as the baseline comparison group in subsequent analysis. The final sample was primarily male (59.73%), non-minority (68.02%), over the age of 40 (86.39%), and with generally high-educational attainment (42.82% holding an associate or bachelor’s degree; 38.73% holding a graduate degree).

**Table 2:** Regression of job dissatisfaction on workplace violence.

Model	Model 1 (IV: Experienced violence) B (SE)	Model 2 (IV: Observed violence) B (SE)	Model 3 (Contrast Model – (Experienced vs. Observed)) B (SE)
Violence Type (per Model)	0.542 (0.084)***	0.427 (0.054)***	0.096 (0.124)
Female (control)	0.021 (0.016)	0.022 (0.016)	0.030 (0.114)
Age 40+ Years (control)	0.024 (0.024)	0.023 (0.024)	−0.451 (0.156)**
Minority Status (control)	−0.039 (0.017)*	−0.038 (0.017)*	−0.176 (0.114)
Education	0.013 (0.011)	0.014 (0.012)	−0.109 (0.076)
Supervisor Status	−0.099 (0.006)***	−0.100 (0.006)***	−0.152 (0.048)**
Tenure	−0.012 (0.008)	−0.012 (0.008)	0.019 (0.057)
Intercept	2.102 (0.039)***	2.100 (0.039)***	3.253 (0.256)***
*F* (df1, df2)	47.148*** (7, 11818)	50.259*** (7, 11,818)	4.384** (7, 351)
*R* ^2^	0.027	0.029	0.080
Δ*F*	42.094***	63.354***	0.607
Δ*R*^2^	0.003	0.005	0.002
*n*	11,826	11,826	359

Unstandardized regression coefficients (B) are reported. Standard errors in parentheses. *p < 0.05, **p < 0.01. ***p < 0.001. Model 3 IV reflects a dichotomous indicator coded as a “1” for those who experienced violence or both experienced and observed violence, and those who only observed violence are coded as a “0”.

### Measures

#### Workplace physical violence

We measured workplace physical violence as a categorical variable reflecting employees physical violence exposure as: (1) experienced—including those with firsthand experience only but also those who report both observing and experiencing physical violence, (2) only observed, and (3) no exposure. *Firsthand experienced physical violence* was measured as physical assault in the workplace within the past two years, assessed using two binary items from the MPS where the anchor stated, “In the past 2 years, have you experienced any acts of workplace violence (e.g., physical assault, threat of assault) that were directed at you?” and the items included: (1) “physical assault that resulted in serious injury” and (2) “physical assault that did not result in serious injury” (U.S. Merit Systems Protection Board, 2016b). Items confirming experience of (3) “threat of assault” or (4) “intentional damage to property in order to intimidate” were excluded. *Observed physical violence* was defined as a third-party witnessing of physical assault directed at others at work during the same timeframe, measured using two items from the same survey where the anchor stated, “In the past 2 years, have you observed any acts of workplace violence that were directed at another person in your workplace” (U.S. Merit Systems Protection Board, 2016b). Items included: (1) “physical assault that resulted in serious injury” and (2) “physical assault that did not result in serious injury” (U.S. Merit Systems Protection Board, 2016b). Items confirming experience of (3) “threat of assault” or (4) “intentional damage to property in order to intimidate” were excluded.

#### Job dissatisfaction

We measured job dissatisfaction using items that reflect job dissatisfaction as a multidimensional construct, consistent with long-standing approaches to the measurement of job satisfaction (Job Descriptive Index approach; Smith et al., 1969; Minnesota Satisfaction Questionnaire; Weiss et al., 1967). Respondents were asked to “please indicate your level of satisfaction or dissatisfaction with the following factors in your current job or work environment” on a Likert-type scale where “1” indicated “very dissatisfied” and a “5” indicated “very satisfied” (U.S. Merit Systems Protection Board, 2016b). The items include: (1) “interesting work that I enjoy”; (2) “feeling respected by colleagues/supervisors/managers”; (3) “opportunity to exercise job-related expertise and judgment”; and (4) “job security” (U.S. Merit Systems Protection Board, 2016b). The items were reverse-scored, reflecting job dissatisfaction. The four-item scale demonstrated good internal consistency (*α* = 0.818).

#### Perceived lack of organizational violence prevention

Perceived lack of organizational violence prevention (LOVP) was measured using four MPS items reflecting organizational prevention efforts of (1) workplace violence; (2) workplace aggression; (3) workplace non-sexual harassment; and (4) workplace sexual harassment. Respondents were asked to indicate agreement with the following statement: “My agency takes sufficient steps to prevent …” on a Likert-type scale, where a “1” indicates strong disagreement and a “5” indicates a strong agreement of (1) “workplace violence from occurring”; (2) “workplace aggression/bullying that is not related to legally protected bases”; (3) “harassment based on legally protected bases other than sex (e.g. race, age, disability) from occurring at my workplace”; (4) “sexual harassment” (U.S. Merit Systems Protection Board, 2016b). The items were reverse scored to indicate LOVP. The scale demonstrated strong internal reliability (α = 0.920).

#### Control variables

Consistent with similar methodological approaches (i.e., Breevaart et al., 2015) and prior research in the transgression literature (Fincham et al., 2005; Stackhouse et al., 2024) we included several demographics (i.e., age, education, gender, and ethnicity) and work-related (i.e., tenure, job code) background variables in our models.

### Analytical method

To test the hypotheses, we used a combination of regression analysis and structural equation modeling. First, direct and contrast hypotheses comparing experienced and observed workplace physical violence (H1a–H1c) were tested using regression analyses conducted in R (version 4.4.1), with job dissatisfaction modeled using mean-averaged scale scores. These analyses estimated total effects and formal contrasts between exposure types.

Second, mediation hypotheses (H2–H3) were tested using structural equation modeling (SEM) in R (lavaan, version 0.6–21). In these analyses, perceived lack of organizational violence prevention (LOVP) and job dissatisfaction were modeled as latent constructs indicated by their respective survey items, allowing explicit modeling of measurement error. The SEM framework enabled simultaneous estimation of direct, indirect, and total effects involving experienced and observed workplace violence while accommodating categorical predictors and non-normal indirect-effect distributions.

Third, Hypotheses 4a–4c were tested by estimating and directly comparing the indirect effects of experienced and observed workplace violence on job dissatisfaction via LOVP within the same SEM. Differences between indirect effects were evaluated using nonparametric bootstrapping with 5,000 resamples to obtain bias-corrected standard errors and confidence intervals (MacKinnon et al., 2007; Preacher and Hayes, 2008).

## Results

### Descriptive statistics

Means, standard deviations, correlations, and internal consistencies of study variables appear in Table 1. We expected direct experience of physical workplace violence and observed physical violence to relate positively with LOVP and job dissatisfaction. As expected, both positively associated with LOVP (*r* = 0.059, *p* = <0.001; *r* = 0.077, *p =* <0.001, respectively) and job dissatisfaction (*r* = 0.039, *p* = <0.001; *r* = 0.050, *p* < 0.001, respectively) as employees who reported either experiencing or observing violence were also slightly more likely to perceive organizational shortcomings in violence prevention and report greater dissatisfaction.

**Table 1:** Descriptive statistics and correlations.

Variable	% Yes/Mean	SD	1	2	3	4	5	6	7	8	9	10
1. Gender (% Female)	40.272%	—										
2. Age (% 40 + years)	86.388%	—	−0.021*									
3. Ethnicity (% Minority)	31.976%	—	0.103*	−0.019								
4. Education	—	—	−0.047*	−0.053*	−0.085*							
5. Job code	—	—	−0.111*	0.153*	−0.077*	0.135*						
6. Tenure	—	—	0.069*	0.346*	−0.011	−0.096*	0.204*					
7. Experienced WPV	0.905%	—	0.011	−0.023*	0.037*	−0.025*	−0.005	−0.012				
8. Observed WPV	2.197%	—	−0.001	−0.014	0.026*	−0.018*	0.007	−0.012	−0.014			
9. LOVP	2.029	0.901	0.116*	−0.016	0.047*	−0.008	−0.108*	−0.010	0.059*	0.077*	0.920	
10. Job dissatisfaction	1.864	0.849	0.013	−0.031*	−0.026*	−0.008	−0.111*	−0.050*	0.039*	0.050*	0.344*	0.818

Correlations are Kendal-Tau for binary-ordinal or ordinal-ordinal pairs and Pearson for binary-binary. Diagonal values represent Cronbach’s alpha for multi-item scales. Gender coded as 1 = female and 0 = male; Age coded as 1 = 40 + years and 0 = 39 years or younger. Ethnicity coded as 1 = minority and 0 = non-minority; Education is an ordinal variable where 1 = less than associate’s college degree (i.e., less than high school diploma, high school/equivalent diploma/GED, some college credits but no degree); 2 = associate’s college degree or bachelor’s college degree; 3 = graduate degree (i.e., master’s degree, professional degree, academic or scientific doctorate). Job code is an ordinal variable where 1 = non-supervisor; 2 = team leader; 3 = supervisor; 4 = manager; 5 = executive. Experienced is coded as 1 = experienced workplace violence and 0 = did not experience or observe workplace violence; observed workplace violence is coded as 1 = observed workplace violence and 0 = did not observe workplace violence. LOVP and job dissatisfaction reflect composite variables. Correlations calculated using listwise deletion. *p < 0.05.

### Robustness checks for common method bias

To assess the potential influence of common method bias, we conducted two diagnostic analyses prior to hypothesis testing. First, we estimated a constrained latent method factor model in which all method factor loadings were fixed to 1.0 to provide a stable and uniform estimate of shared method variance (Williams et al., 1989; Williams and McGonagle, 2016). Inclusion of the method factor resulted in only trivial improvements in overall model fit relative to the baseline model (*Δ*χ^2^ = −74.2116; *ΔCFI* = 0.0014; *ΔRMSEA* = −0.0007; Δ*SRMR* = −0.0002; Δ*TLI* = 0.0012), and all indirect effects remained statistically significant and substantively unchanged. Second, recognizing ongoing debates about the utility of unmeasured latent method factors (Podasakoff et al., 2003; Richardson et al., 2009), we conducted a marker variable analysis (Podsakoff et al., 2012). The marker factor, reflecting difficulty in the job search process, showed small but statistically significant correlations with LOVP (*r* = 0.140) and job dissatisfaction (*r* = 0.129), accounting for less than 2% of shared variance in each case. These values fall well below thresholds typically associated with substantive method bias (Lindell and Whitney, 2001; Podasakoff et al., 2003), suggesting that common method bias does not meaningfully threaten interpretation of the results.

### Statistical models

Direct, indirect, and total effects were estimated using 5,000 bootstrap resamples to address non-normality in the indirect-effect distributions. The model included experienced and observed workplace physical violence as predictors, perceived lack of organizational violence prevention (LOVP) as the mediator, and job dissatisfaction as the outcome. As a preliminary diagnostic, mediated model fit was evaluated and found to be acceptable (*CFI* = 0.969, *RMSEA* = 0.049, *SRMR* = 0.019, *TLI* = 0.957; *χ*^2^ = 1748.13, *p* < 0.001).

### Results for direct contrast hypotheses (H1a-c)

Regression results are presented in Table 2. We compared job dissatisfaction between employees who experienced physical workplace violence and those who observed it. The total effect of experienced violence on job dissatisfaction was *B* = 0.542, *SE* = 0.084, *p* < 0.001, while the total effect of observed violence was *B* = 0.427, *SE* = 0.054, *p* < 0.001. Although the point estimate for experienced violence was larger, the difference between the two effects was nonsignificant (*Δ* = 0.096, *SE* = 0.124, *p* = 0.436). Thus, neither Hypothesis 1a nor Hypothesis 1b was supported; instead, results align with Hypothesis 1c, indicating no significant difference in job dissatisfaction between employees who directly experienced violence and those who witnessed it. Both exposure types similarly undermined job attitudes.

### Results for mediation and indirect contrasts (H2-H4)

Results of the mediation analyses are reported in Table 3. Hypothesis 2 examined the explanatory mediating role of LOVP following experienced physical workplace violence on job dissatisfaction. The direct effect was nonsignificant (*B* = 0.144, *SE* = 0.084, *p* = 0.087). However, experiencing physical violence predicted LOVP (*B* = 0.805, *SE* = 0.121, *p* < 0.001), which predicted job dissatisfaction (*B* = 0.409, *SE* = 0.013, *p* < 0.001). As such, the indirect effect was significant (*B* = 0.329, *SE* = 0.050, *p* < 0.001).

**Table 3:** Summary of effects of violence on job dissatisfaction through perceived lack of organizational violence prevention.

Indirect, direct and total effects	*B* (*SE*)	*P*	95% Confidence Interval	
			*LB*	*UB*
Indirect effects				
Experienced violence > LOVP > Job dissatisfaction	0.329 (0.050)	<0.001	0.229	0.431
Observed violence > LOVP > Job dissatisfaction	0.247 (0.029)	<0.001	0.190	0.307
Direct effects				
Experienced violence > Job dissatisfaction	0.144 (0.084)	0.087	−0.026	0.310
Observed violence > Job dissatisfaction	0.084 (0.046)	0.067	−0.006	0.172
Total effects				
Experienced violence > Job dissatisfaction	0.473 (0.093)	<0.001	0.291	0.657
Observed violence > Job dissatisfaction	0.331 (0.055)	<0.001	0.224	0.438
Contrast effects				
Difference in indirect effects for Exp. vs. Obs. violence	0.082 (0.057)	0.151	−0.030	0.195
Difference in total effects for Exp. vs. Obs. violence	0.143 (0.107)	0.182	−0.072	0.348

Unstandardized regression coefficients (B) are reported. Standard errors are shown in parentheses. Confidence intervals were estimated with the percentile bootstrap method and 5,000 bootstrap resamples. Lower bound (LB) reflects the 2.5th percentile and upper bound (UB) reflects 97.5th percentile of the bootstrap distribution. “LOVP” reflects perceived Lack of Violence Prevention.

Hypothesis 3 posited that LOVP mediates the relationship between observing physical workplace violence and job dissatisfaction. The direct effect was nonsignificant (*B* = 0.084, *SE* = 0.046, *p* = 0.067). But, observing physical violence predicted LOVP (*B* = 0.604, *SE* = 0.070, *p* < 0.001), which predicted job dissatisfaction (*B* = 0.409, *SE* = 0.013, *p* < 0.001). Thus, the indirect effect was significant (*B* = 0.247, *SE* = 0.029, *p* < 0.001).

Hypothesis 4a-c examined whether the indirect effect of workplace physical violence on job dissatisfaction via LOVP differed by exposure type. As stated above, both indirect effects were significant: experienced violence to LOVP to job dissatisfaction: (*B* = 0.329, *SE* = 0.050, *p* < 0.001); observed violence to LOVP to job dissatisfaction: (*B* = 0.247, *SE* = 0.029, *p* < 0.001). The difference between these indirect effects was nonsignificant (Δ = 0.082, *SE* = 0.057, *p* = 0.151), indicating that the mediated impact of experienced and observed violence did not differ. Thus, neither Hypothesis 4a nor Hypothesis 4b was supported; results align with Hypothesis 4c, consistent with the theoretical expectation that prevention perceptions, not proximity to harm, drive job dissatisfaction.

## Discussion

Grounded in Psychological Contract Theory and Social Exchange Theory (Blau, 1964/1986; Robinson and Rousseau, 1994), our results suggest that violence, whether witnessed or endured, functions as a relational signal of breach. When prevention efforts appear inadequate, employees infer neglect of the implicit safety obligation, recalibrating their evaluations of the employer and withdrawing trust. Thus, the observed equivalence between firsthand and vicarious third-party exposure reframes workplace violence as an organizational failure rather than a stressor contingent on personal victimization, advancing a relational interpretation of harm.

The present study contributes to theory by clarifying how workplace physical violence shapes employee job attitudes and by specifying the organizational mechanisms through which that influence occurs. Rather than treating violence as a purely individual stressor whose consequences depend on proximity to harm, our findings point to a relational and organizational account centered on how employees evaluate organizational responsibility for safety. Below, we interpret our results in light of existing theories and delineate how they refine core assumptions in workplace violence, psychological contract, and organizational evaluation research.

The present findings challenge conventional assumptions in the workplace violence literature (Johnson and O’Leary-Kelly, 2003; May and Wisco, 2016; Michael et al., 2016; Zhou et al., 2017) by showing that job dissatisfaction does not differ significantly between employees who directly experience physical violence and those who observe it. Although stress-based perspectives have long emphasized proximity to harm as the primary driver of work-related consequences (Barling, 1996; Michael et al., 2016), our results indicate that experiential immediacy is less decisive for evaluative job attitudes than prior theory suggests. Both experienced and observed violence were associated with significant increases in job dissatisfaction, and the contrast between the two exposure types was negligible. This pattern suggests that job dissatisfaction reflects not only personal reactions to harm, but broader judgments about the employment relationship that are activated by violence irrespective of direct victimization.

Importantly, these findings point to a limitation of traditional stressor–strain models in explaining work attitudes following high-intensity events. While proximity to violence is likely to shape fear, physiological arousal, and posttraumatic responses (Dupré et al., 2014; Zhou et al., 2017), our findings show no substantive difference between being the object of violence and the witness of violence in how satisfied someone is with their job (Harris et al., 2013). What this points to is that evaluative outcomes such as job dissatisfaction appear to be governed by interpretive processes that extend beyond individual stress responses. Observers are not passive or unaffected by violence they witness (Miner and Cortina, 2016). Rather, they engage in organizational sensemaking, drawing conclusions about whether their employer has upheld fundamental obligations related to safety and protection. In this respect, our findings builds on emerging research on severe mistreatment observers (Wilkerson and Meyer, 2019), which demonstrates that witnessing harm can meaningfully erode trust, justice perceptions, and attachment to the organization (Dhanani and LaPalme, 2019; Hill et al., 2025).

The mediating role of perceived lack of organizational violence prevention provides critical insight into *why* both experienced and observed violence undermine job satisfaction. When perceptions of prevention were taken into account, the direct effects of violence exposure on job dissatisfaction became non-significant, indicating that employees’ evaluative responses are not driven by the violent act itself but by how that act is interpreted in relation to organizational responsibility. This pattern suggests that physical violence serves as an initial cue that draws attention to the organization’s safety obligations, but that dissatisfaction crystallizes only when employees infer that the organization failed to anticipate, prevent, or adequately manage the risk of harm. In this respect, violence functions less as a discrete stressor and more as a diagnostic signal of organizational neglect, prompting employees to reassess whether their employer has upheld its most fundamental obligations (Rousseau, 1995; Robinson, 1996; Walker and Hutton, 2006; Walker, 2013).

This finding advances psychological contract theory by clarifying how breach judgments emerge in the context of workplace violence. Psychological contract research has long emphasized that breach is not triggered by negative events per se, but by employees’ interpretations of whether those events reflect failures to fulfill implicit obligations (Morrison and Robinson, 1997; Robinson and Rousseau, 1994). Our results specify that, in the case of physical violence, perceptions of prevention constitute the primary basis for those interpretations. Rather than responding solely to the occurrence of harm, employees appear to evaluate whether the organization took reasonable and visible steps to uphold its duty of care before, during, and after the incident. When prevention efforts are perceived as credible and consistently enforced, violence may be construed as an unfortunate but unrepresentative deviation. When prevention is perceived as lacking, however, the same event is reinterpreted as evidence that the organization has failed to honor a core, non-negotiable promise of employment, thereby triggering dissatisfaction and withdrawal-oriented attitudes (e.g., Rousseau, 1995; Walker and Hutton, 2006).

Collectively, the present findings indicate that proximity to harm is not the primary driver of job dissatisfaction when violence is interpreted through organizational prevention failures. Instead, both targets and observers appear to arrive at similar evaluative conclusions because they rely on shared standards regarding organizational responsibility for safety. Psychological contracts are not formed solely through direct interpersonal treatment, but also through collective understandings of what organizations are obligated to prevent and protect against. When prevention is perceived as inadequate, both experienced and observed violence therefore activate comparable breach appraisals, not because observers experience derivative distress, but because they update their judgments about whether the organization can be relied upon to uphold its most basic duty of care (Robinson and Rousseau, 1994; Walker and Hutton, 2006).

This perspective advances psychological contract theory by shifting attention from individual exposure to collective judgment processes (Skarlicki and Kulik, 2005). Prior theorizing has largely treated observers as secondary recipients whose reactions stem from empathy, fear, or vicarious threat (Barling, 1996; Çakır et al., 2025; Schat and Kelloway, 2003). The current findings suggest a different role: observers function as evaluators of organizational integrity, using violent events as information about the organization’s reliability, justice orientation, and commitment to protection (Miner and Cortina, 2016). In this sense, observers’ reactions constitute first-order judgments about the employment relationship rather than spillover responses to others’ harm. This reconceptualization helps explain why proximity to violence does not differentiate job dissatisfaction and underscores that evaluations of organizational breach are anchored in shared standards of prevention and accountability rather than in personal victimization alone (Folger and Cropanzano, 2001; David et al., 2024; Dhanani and LaPalme, 2019).

Our findings also extend social exchange perspectives by demonstrating that exchange evaluations are shaped as much by organizational inaction as by direct treatment. Social exchange theory posits that employees monitor organizational behavior for signals of reciprocity and balance, adjusting their attitudes and behaviors when obligations appear unmet (Blau, 1964/1986; Cropanzano et al., 2017). Perceived failures in violence prevention represent a particularly consequential form of imbalance because they threaten employees’ sense that the organization is willing or able to meet the most basic conditions of exchange. When prevention is lacking, both targets and witnesses infer that the organization is not investing adequately in their safety, prompting reevaluations of the desirability and legitimacy of the employment relationship even in the absence of personal victimization (Parzefall and Salin, 2010).

Taken together, these results encourage a reconceptualization of workplace physical violence as an organizational and relational phenomenon rather than a purely individual stressor. Violence foregrounds questions of organizational governance, foresight, and accountability, inviting collective judgments about whether safety obligations are taken seriously. Observers’ reactions are therefore not collateral consequences of exposure, but central components of how violence reshapes the meaning of work and the employment relationship. The present study demonstrates that job dissatisfaction following extreme workplace violence is driven not by proximity to harm, but by shared evaluations of organizational prevention failure. By showing that perceived lack of organizational violence prevention fully accounts for the effects of both experienced and observed violence, and that differences between exposure types are negligible, this work advances violence research beyond stress-based explanations. It reconceptualizes workplace violence as an organizational judgment problem, in which prevention functions as the mechanism through which extreme events are translated into durable evaluations of the job and the organization.

### Limitations and future research directions

A limitation of the present study is its cross sectional design, which constrains strong causal inference regarding the relationships among workplace violence exposure, perceived lack of organizational violence prevention, and job dissatisfaction (cf. Stone-Romero and Rosopa, 2008). However, this design reflects a necessary and theoretically appropriate trade off associated with studying rare and high severity workplace events. Severe physical workplace violence is low in base rate and ethically constrained, making prospective, longitudinal, or experimental study largely infeasible (Barling, 1996; Dupré et al., 2014; LeBlanc and Kelloway, 2002). Consequently, large scale cross sectional data remain one of the few viable means of capturing employees’ real world exposure to extreme violence and their contemporaneous evaluations of organizational prevention efforts.

The nationally representative sample used here captures real world exposure to high intensity violence across a large workforce, offering rare insight into how such events are interpreted by employees under naturalistic conditions. In this context, the value of the study lies not in establishing temporal causality but in identifying how employees collectively make sense of violence when it occurs and which organizational interpretations are most closely associated with evaluative job attitudes. As such, the cross sectional design is well suited to the paper’s theoretical focus on meaning making, organizational judgment, and relational evaluation, even as it highlights important opportunities for future longitudinal and multi method research.

Importantly, the value of the present design lies not primarily in establishing temporal ordering, but in illuminating the evaluative logic through which extreme physical workplace violence is translated into job attitudes. Theoretical perspectives grounded in psychological contract and social exchange theories emphasize that employees’ perceptions and judgments about organizational responsibility are themselves consequential outcomes, reflecting relatively stable evaluations rather than short-lived affective reactions (Robinson and Rousseau, 1994; Cropanzano et al., 2017; Walker and Hutton, 2006). In the context of high-intensity, rare, and consequential violent events, employees’ contemporaneous assessments of organizational violence prevention and job dissatisfaction therefore provide valid insight into how organizations are judged when fundamental safety expectations are violated, even in the absence of fine-grained temporal sequencing.

From this standpoint, the cross-sectional approach employed here is well aligned with the study’s theoretical focus on organizational meaning making and evaluative judgment under conditions of severe harm. Extreme physical workplace violence represent a qualitatively different category of workplace event, one that foregrounds organizational responsibility in especially stark terms (Barling, 1996; LeBlanc and Kelloway, 2002; Dupré et al., 2014; Stackhouse, 2025), which limits the feasibility of experimental or prospective research designs (Mueller and Tschan, 2011; Stone-Romero and Rosopa, 2008). Precisely because of its intensity, however, it foregrounds core assumptions about organizational duty of care. The present design enables rare empirical access to this class of events at a level of scale and ecological validity that would otherwise be difficult to achieve, offering theoretically valuable insight into how employees interpret and evaluate organizational responsibility in the aftermath of serious workplace violence.

Future research should examine whether the prevention-based evaluative processes identified here are specific to *extreme physical workplace violence* or extend to lower-intensity but more chronic forms of mistreatment, such as incivility, bullying, or social undermining. Prior research has often treated workplace aggression as a continuum, emphasizing differences in frequency rather than differences in kind (Barling, 1996; Hershcovis, 2011). However, extreme physical violence may constitute a qualitatively distinct class of event that heightens moral salience and renders organizational safety obligations especially salient, thereby anchoring employee evaluations in judgments of fundamental duty of care (Schat and Kelloway, 2000, 2003). In contrast, lower-intensity mistreatment, while often more pervasive, may be interpreted through alternative evaluative standards, such as norms of respect, fairness, or interpersonal justice, rather than through prevention-based breach.

Comparative examination of these event types would therefore advance theory by clarifying whether perceived prevention failure operates as a universal organizational accountability signal or as a boundary-conditioned mechanism activated primarily by severe, morally consequential harm. Although prior work demonstrates that both personal and group-level exposure to lower-intensity mistreatment can undermine job attitudes (Andersson and Pearson, 1999; Lim et al., 2008), it remains unclear whether similar mechanisms of organizational judgment are engaged when harm does not implicate physical safety. By explicitly contrasting extreme violence with more common forms of mistreatment, future research could specify when organizational prevention becomes the dominant lens for evaluation and when other meaning-making processes govern employee responses. This line of inquiry would refine violence and mistreatment theories by moving beyond severity as a matter of degree and toward a more precise understanding of how different forms of harm activate distinct organizational judgment systems.

Although the present study demonstrates convergence in evaluative judgments following experienced and observed violence, future research should examine the range of behavioral responses that may follow from these shared judgments. Prevention failures may motivate corrective behaviors such as voice, withdrawal, or organization-directed harm, depending on perceived accountability, feasibility of action, and organizational entitativity. Building on displaced revenge research showing that harm is often redirected toward organizations when responsibility is collective (Stackhouse et al., 2023), future work could theorize how prevention-based judgments shape response selection rather than response intensity.

Finally, future research should examine whether and how organizations can repair prevention-based breach following extreme workplace violence. The present findings indicate that job dissatisfaction reflects employees’ judgments that the organization failed to uphold its most fundamental obligation to ensure physical safety. This raises an important theoretical question about whether such prevention-based breach appraisals are fixed once formed, particularly following extreme and morally salient events, or whether they remain responsive to subsequent organizational action. Prior research on repair and reconciliation has largely focused on interpersonal wrongdoing, emphasizing processes such as apology, forgiveness, and trust restoration in dyadic relationships (Aquino et al., 2006; Bies and Tripp, 2005). Extreme workplace violence, however, shifts the locus of responsibility from individual perpetrators to the organization itself, creating a qualitatively different repair challenge.

Future work could therefore extend repair and reconciliation theories by examining prevention as a central mechanism of organizational moral repair. Visible post-incident responses, transparent accountability for safety failures, and credible investments in prevention may serve as institutional signals of renewed commitment, potentially attenuating breach appraisals even after severe harm has occurred (Walker and Hutton, 2006; Walker, 2013). Such actions may foster forgiveness or benevolence not by negating the violence itself, but by reshaping employees’ interpretations of organizational intent, reliability, and moral standing. Consistent with recent work on group betrayal and displaced responses, organizational efforts to acknowledge and address prevention failures may play a critical role in determining whether employees seek retribution, forgive (or not), withdraw, or re-engage with the organization following harm (Stackhouse et al., 2023). Examining these processes would advance theory by moving beyond interpersonal accounts of repair to specify how institutions attempt to reconstitute the employment relationship in the aftermath of extreme violence understood as both an organizational failure and a broader ethical violation.

### Practical implications

This research offers several practical implications for organizations navigating the complexities of workplace violence. Existing scholarship and organizational practice have primarily emphasized three areas of violence prevention and response: post-incident support for direct victims, behavioral management and accountability mechanisms for aggressors, and system-level interventions aimed at monitoring and prevention (e.g., Sammut et al., 2025). Our findings extend this work by demonstrating that the consequences of workplace violence are not confined to those who are directly harmed. Observers reported levels of job dissatisfaction comparable to those of direct victims, suggesting that violent incidents shape organizational evaluations across the broader workforce. Accordingly, response protocols should not be limited to victim-focused interventions alone. Managers and HR professionals may need to assume that violent incidents reverberate through work units and proactively engage the wider employee population through transparent communication, reassurance, and visible accountability measures. Addressing both direct and indirect exposure may help organizations more effectively sustain employee attitudes following violent events.

Second, organizations should invest in preventive infrastructure for workplace violence. Existing prevention programs often evaluate effectiveness primarily in terms of incident reduction, offering limited insight into how preventive efforts shape employees’ perceptions of safety or job attitudes (Sammut et al., 2025). Our findings suggest that violence undermines employee attitudes primarily when incidents are interpreted as failures of organizational prevention. This highlights the importance of not only reducing workplace violence, but also ensuring that preventive efforts are visible, credible, and perceived as effective by employees.

From a psychological contract perspective, findings indicate that workplace violence prompts employees to evaluate whether the organization has violated a core, nonnegotiable obligation to ensure safety. Importantly, this evaluative process is not limited to direct victims; observers similarly interpret violent incidents as diagnostic of the employer’s fulfillment of its responsibilities. As a result, violence threatens relational trust even when harm is not personally experienced. Leaders should therefore anticipate that such incidents carry collective consequences for employee attitudes. Proactive psychological contract management, including the articulation of clear safety expectations, timely acknowledgment of failures, and explicit recommitment to prevention, may help mitigate dissatisfaction. Ultimately, organizational responses must move beyond empathy or procedural compliance to clearly reaffirm the organization’s responsibility for safety and its commitment to preventing future harm.

## Conclusion

This study demonstrates that workplace violence carries organizational consequences that extend well beyond direct victimization. Experienced and observed violence were similarly associated with job dissatisfaction, and in both cases this relationship was fully explained by perceived failures in organizational violence prevention. These findings challenge assumptions that firsthand exposure is uniquely damaging and instead highlight how violence serves as a collective signal about organizational responsibility and safety. Employees appear to evaluate violent incidents not as isolated acts, but as evidence of whether the organization has upheld a fundamental, nonnegotiable obligation to protect its workforce. By reframing workplace violence as a relational and organizational failure rather than a purely individual stressor, this research advances theory and underscores the importance of visible, credible prevention efforts for sustaining employee trust and attitudes.

## Data Availability

Publicly available datasets were analyzed in this study. This data can be found here: https://catalog.data.gov/dataset/merit-principles-survey-2016-data.
